# Gene Variations in *Cis*-Acting Elements between the Taiwan and Prototype Strains of Porcine Epidemic Diarrhea Virus Alter Viral Gene Expression

**DOI:** 10.3390/genes9120591

**Published:** 2018-11-29

**Authors:** Tsung-Lin Tsai, Chen-Chang Su, Ching-Chi Hsieh, Chao-Nan Lin, Hui-Wen Chang, Chen-Yu Lo, Ching-Houng Lin, Hung-Yi Wu

**Affiliations:** 1 Graduate Institute of Veterinary Pathobiology, College of Veterinary Medicine, National Chung Hsing University, Taichung 40227, Taiwan; windtaker10@msn.com (T.-L.T.); bluesnoteguitar@gmail.com (C.-C.S.); axfbji7917@gmail.com (C.-Y.L.); tw23whale@hotmail.com (C.-H.L.); 2 Division of Chest Medicine, Department of Internal Medicine, Chang Bing Show Chwan Memorial Hospital, Changhua 505, Taiwan; mycatti@gmail.com; 3 Department of Veterinary Medicine, National Pingtung University of Science and Technology, Neipu, Pingtung 91201, Taiwan; cnlin6@gmail.com; 4 Graduate Institute of Molecular and Comparative Pathobiology, School of Veterinary Medicine, National Taiwan University, Taipei 10617, Taiwan; huiwenchang@ntu.edu.tw

**Keywords:** porcine epidemic diarrhea virus, *cis*-acting element, gene evolution, gene expression, nucleotide composition

## Abstract

In 2013, the outbreak of porcine epidemic diarrhea (PED) in Taiwan caused serious economic losses. In this study, we examined whether the variations of the *cis*-acting elements between the porcine epidemic diarrhea virus (PEDV) Taiwan (TW) strain and the prototype strain CV777 alter gene expression. For this aim, we analyzed the variations of the *cis*-acting elements in the 5′ and 3′ untranslated regions (UTRs) between the PEDV TW, CV777, and other reference strains. We also determined the previously unidentified transcription regulatory sequence (TRS), a sequence motif required for coronavirus transcription, and found that a nucleotide deletion in the TW strain, in comparison with CV777 strain, immediately downstream of the leader core sequence alters the identity between the leader TRS and the body TRS. Functional analyses using coronavirus defective interfering (DI) RNA revealed that such variations in *cis*-acting elements for the TW strain compared with the CV777 strain have an influence on the efficiency of gene expression. The current data show for the first time the evolution of PEDV in terms of *cis*-acting elements and their effects on gene expression, and thus may contribute to our understanding of recent PED outbreaks worldwide.

## 1. Introduction

Porcine epidemic diarrhea virus (PEDV) belongs to the genus *Alphacoronavirus* of subfamily *Coronavirinae*, which is in the family *Coronaviridae*, order *Nidovirales* [1,2]. The capped PEDV genome of ≈28 kilobases consists of a 5′ untranslated region (UTR), open reading frames (ORFs), and a 3′ UTR that includes the poly(A) tail [3]. RNA elements in the 5′ and 3′ untranslated regions (UTRs) that are important for coronaviral gene expression are collectively referred to as *cis*-acting RNA elements [4]. For the coronavirus, the transcription regulatory sequence (TRS) is a *cis*-acting RNA element required for subgenomic mRNA (sgmRNA) synthesis that is located downstream of the leader sequence and upstream of each ORF in the 3′-proximal genome region [5]. An upstream open reading frame (uORF) located in the 5′ UTR of coronaviruses has been shown to be important for virus fitness [6].

Analyses of *Alphacoronavirus* 5′ UTRs have revealed four conserved RNA secondary structures, including stem-loops (SLs) 1, 2, 4, and 5, which are also conserved for *Betacoronavirus* [4,7]. Moreover, studies in *Betacoronavirus* have determined a requirement of these 5′ secondary structures for coronaviral RNA synthesis [8]. RNA structures and their functions in coronaviral RNA synthesis and pathogenesis have also been identified in *Betacoronavirus* 3′ UTRs, including the 5′-most bulged stem-loop (BSL) [9,10], hair-pin pseudoknot (PK) [11], and 3′-most hypervariable region (HVR) [12]. However, structural analyses by Madhugiri et al. have suggested the absence of a BSL equivalent in *Alphacoronavirus* [4]. Both *Alphacoronavirus* and *Betacoronavirus* contain the conserved octanucleotide sequence 5′-GGAAGAGC-3′ within the HVR [4].

Synthesis of sgmRNA in coronaviruses requires a conserved TRS sequence motif, which is located at the 3′ end of the leader sequence (TRS-L) and precedes each 3′ proximal gene (TRS-B). The TRS contains a conserved sequence (CS) flanked by variable sequences at its 5′ (5′ TRS) and 3′ (3′ TRS) ends [5,13]. During negative-strand sgmRNA synthesis, coronaviral polymerase along with negative-strand sgmRNA attenuates at the TRS-B, switches the template to the TRS-L, as guided by basepairing between the TRS-B and TRS-L, and then resumes to complete synthesis by copying the leader sequence [14,15]. In comparison with the 5′ TRS, the 3′ TRS has a more decisive influence on sgmRNA synthesis [16,17]. It has been proposed that a one-nucleotide (nt) (A) deletion immediately downstream of the CS-L may influence PEDV sgmRNA synthesis [3,18,19], though an in-depth examination to support this hypothesis has not been conducted.

The PEDV prototype strain CV777 was first identified in 1978, and suckling piglets infected with this strain exhibited only mild diarrhea [20,21,22]. In 2013, the sudden appearance of porcine epidemic diarrhea (PED) with more severe diarrhea in the USA and a subsequent reoccurrence in Asian countries including Taiwan caused serious economic losses [3,23,24]. PED has since been one of the most devastating swine diseases worldwide. With such outbreaks, a crucial question is whether the pathogenicity of PEDV has been altered, leading to serious disease outbreaks. Because the coronavirus spike (S) protein interacts with a cellular receptor during virus entry [25,26] and functions by inducing neutralizing antibodies in the natural host [27,28,29], many studies have focused on a sequence comparison of the S protein between emergent or re-emergent PEDV strains and the prototype strain CV777 to seek a possible answer for PED outbreaks [18,24,30,31]. In addition to S protein, whether other factors play roles in the outbreak remain to be elucidated.

The evolution of *cis*-acting elements in PEDV has not been previously analyzed. In this study, we determined whether the *cis*-acting elements that are required for gene expression are altered in the PEDV Taiwan (TW) strain in comparison with those of the PEDV prototype strain CV777. Functional analyses were also performed to determine whether the identified variations in *cis*-acting elements between the two strains alter the efficiency of translation, replication (genomic RNA synthesis), and transcription (sgmRNA synthesis) of the virus. The results may extend our understanding for the recent PED outbreaks in Taiwan and other countries.

## 2. Materials and Methods

### 2.1. Sample Collection

Fecal and intestinal samples were collected from the Animal Disease Diagnostic Center of National Chung Hsing University and National Pingtung University of Science and Technology between January 2014 and December 2016. The total sample size was 38. Samples were homogenized, and RNA was extracted using TRIzol (Thermo Fisher Scientific, Waltham, MA, USA) according to the manufacturer’s instructions.

### 2.2. Amplification of the Genome and sgmRNA Using RT-PCR and Sequencing Analyses

Random hexamer primers were used for reverse transcription (RT) with SuperScript III reverse transcriptase (Invitrogen, Carlsbad, CA, USA), and the resulting cDNA was used for PCR with PfuUltra II high-fidelity DNA polymerase (Agilent, Santa Clara, CA, USA) and oligonucleotides PEDV 25(−) and PEDV 450(+) for the 5′ UTR and PEDV 27488 (−) and PEDV 3′end (+) for the 3′ UTR (Table S1). The resulting 50-µL PCR mixture was heated to 94 °C for 2 min and subjected to 34 cycles of 30 s at 94 °C, 30 s at 55 °C, and 30 s at 72 °C. The extreme 5′ and 3′ termini of the PEDV genome were identified via rapid amplification of cDNA ends (RACE) (Thermo Fisher Scientific) according to the manufacturer’s instructions. To amplify each of the sgmRNA for identification of the core sequence, oligonucleotide PEDV 25(−) and a corresponding reverse primer specific for each sgmRNA (Table S1) were used, followed by sequencing analyses. To identify the sgmRNA derived from PEDV 3′ UTR, oligonucleotides PEDV 25(−) and PEDV 3′utr (+) were used. The resulting 50-µL PCR mixture was heated to 94 °C for 2 min and subjected to 30 cycles of 30 s at 94 °C, 30 s at 55 °C, and 90 s at 72 °C.

### 2.3. Plasmid Constructs

To construct the PEDV TW defective interfering (DI) RNA pTWDI, an overlap PCR mutagenesis procedure was performed, as previously described [32], but with oligonucleotides T7-PEDV5′UTR(−) and PEDV26696(+), and PEDV TW strain cDNA in the first PCR; oligonucleotides PEDV 26541 (−) and PEDV3′end (+), and PEDV TW strain cDNA in the second PCR; and oligonucleotides T7-PEDV 5′UTR(−) and PEDV 3′end (+), and the products of the first two reactions in a third PCR. The resulting PCR product was then cloned into the TOPO XL vector (Thermo Fisher Scientific). To construct CV777 DI RNA, in which both the 5′ and 3′ UTR were from PEDV CV777 but the remaining sequences were from PEDV TW, pTWDI was used as a template; the aforementioned overlap PCR mutagenesis procedure was also performed with mutation oligonucleotides listed in Table S1. Mutants pTWDI-EP, pCV777DI-EP, pΔ AS-TRS, and pS-TRS were similarly constructed, except for the corresponding oligonucleotides used in the first and second reactions, as described in Table S1.

### 2.4. Analysis of DI RNA and sgm DI RNA Synthesis Using RT-qPCR

The PEDV TW strain (GenBank Accession No. KP276252) was obtained from Dr. Hui-Wen Chang (National Taiwan University) [33] and used as the helper virus for the DI RNA replication assay. The PEDV TW strain was maintained in Vero cells, as previously described [34,35]; the viral titer was 10^5^ TCID50/mL. Vero cells in 35-mm dishes were infected with 200 µL of 10^5^ TCID50/mL of PEDVPT-P5. The DNA constructs pTWDI, pCV777DI, pΔA S-TRS, and pS-TRS were linearized with *Mlu*I, transcribed in vitro to synthesize RNA transcripts with the mMessage mMachine T7 transcription kit (Thermo Fisher Scientific) according to the manufacturer’s instructions and passed through a Biospin 6 column (Bio-Rad, Hercules, CA, USA). After 2 h of infection with the helper virus, 3 µg of RNA transcript was transfected into the cells. To detect replication of PEDV DI RNA and DI RNA-derived sgmRNAs (sgm DI RNA), the supernatant was collected at 48 hour posttransfection (hpt) and then used to infect fresh Vero cells (virus passage 1, VP1). Ten micrograms of TRIzol-extracted total cellular RNA at 48 hpi of VP1 was used for the RT reaction with the reporter oligonucleotide TGEV(+) and SuperScript III reverse transcriptase (Thermo Fisher Scientific). To examine synthesis of DI RNA, SYBR^®^ green amplification mix (Roche Applied Science, Mannheim, Germany) and primers PEDV476(−) and TGEV(+) were used for qPCR according to the manufacturer’s protocol. To analyze the synthesis of sgm DI RNA, primers PEDVL20(−), which binds to the leader sequence, and 5′GD(+), which binds to the reporter gene, were employed to differentiate the DI RNA from the helper virus PEDV. In these experiments, dilutions of plasmids containing the same gene as the detected DI RNA or sgm DI RNA were always run in parallel with the quantitated cDNA for use in standard curves (dilutions ranged from 10^8^ to 10 copies of each plasmid). The amount of synthesized RNA was normalized to the levels of internal controls, including helper virus genomic RNA, 18S rRNA, and DI RNA. The reactions were performed with an initial pre-incubation at 95 °C for 5 min, followed by 35 amplification cycles of 95 °C for 15 s and 60 °C for 60 s.

### 2.5. Western Blot Analysis for DI RNA Translation in Cells

The DNA constructs pTWDI-EP and pCV777DI-EP were linearized with *Mlu*I, transcribed in vitro with the mMessage mMachine T7 transcription kit (Thermo Fisher Scientific) according to the manufacturer’s instructions, and passed through a Biospin 6 column (Bio-Rad). Vero cells were then transfected with 3 µg of transcript RNA, and proteins in cell lysates were collected at 16 h posttransfection. The protein samples were separated using 12% sodium dodecyl sulphate-polyacrylamide gel electrophoresis (SDS-PAGE) gels and electrotransferred onto nitrocellulose membranes (GE Healthcare, Chicago, IL, USA). An antibody against enhanced green fluorescent protein (EGFP) was used as the primary antibody; goat anti-mouse IgG conjugated to horseradish peroxidase (HRPO) was the secondary antibody (Jackson Laboratory, Bar Harbor, ME, USA). Detected EGFP was visualized using Western Lightning™ Chemiluminescence Reagent (Perkin Elmer, Waltham, MA, USA) and X-ray film (Kodak, Rochester, NY, USA).

### 2.6. Statistical Analysis and Sequence Alignment

Statistical analysis of the data was performed with Student’s unpaired *t*-test using Prism 6.0 software (GraphPad Software, La Jolla, CA, USA). Values are presented as the mean ± standard deviation (SD) (*n* = 3); * *p* < 0.05, ** *p* < 0.01, and *** *p* < 0.001. The MegAlign program (DNASTAR, Madison, WI, USA) was used for the alignment of nucleotide and deduced amino acid sequences.

## 3. Results and Discussion

### 3.1. Comparison of Sequence and Structure in the 5′ UTR between CV777 and TW Strain

**Figure 1:**
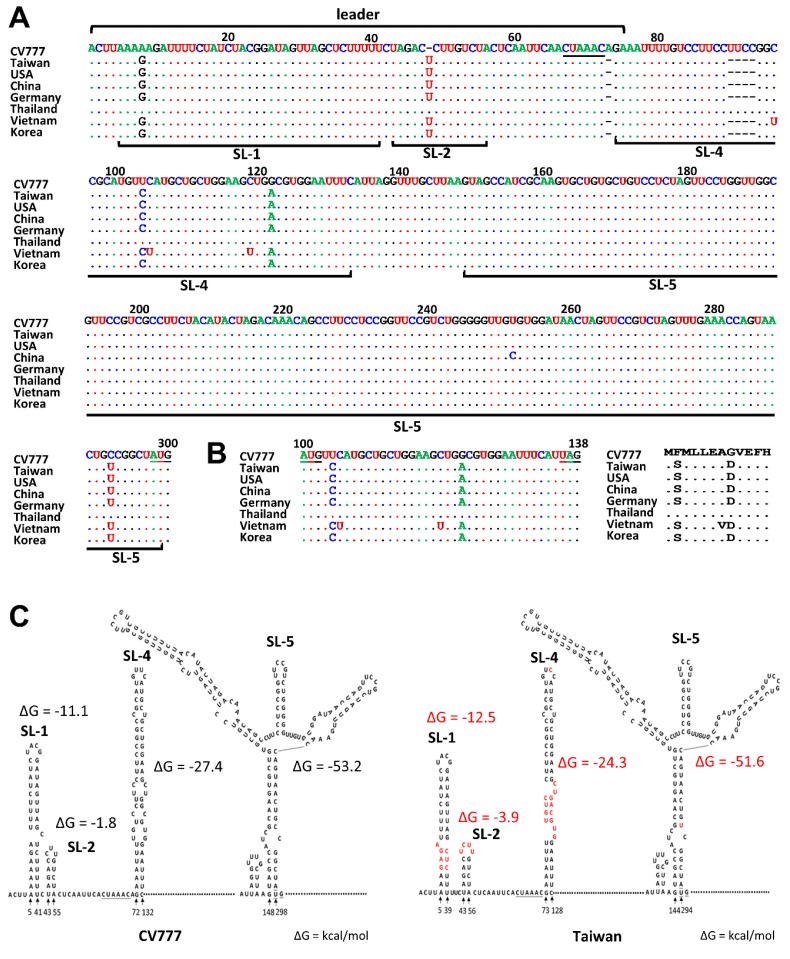
Sequences and structures of the porcine epidemic diarrhea virus (PEDV) 5′ untranslated region (UTR). (**A**) Nucleotide sequence alignment of the 5′ UTR between CV777, Taiwan (TW), and reference strains. The position numbers refer to the composite sequence. Nucleotide identity between sequences is indicated with a dot; a difference is indicated with a new letter code. A gap is indicated with a dash. Underlining indicates the core sequence of the leader (CS-L) (nt 67–72) and the start codon for ORF 1a (nt 298–300). (**B**) Alignment of the nucleotide sequence (left panel) and deduced amino acid sequence (right panel) of the upstream open reading frame (uORF) for CV777, TW, and reference strains. (**C**) Structure prediction of the 5′ UTR of strains CV777 (left panel) and TW (right panel). The CS-L and start codon are indicated with underlining. CV777 (GenBank accession number AF_353511); USA (GenBank accession number KF_468572); China (GenBank accession number KC_210145); Germany (GenBank accession number LM_645058); Thailand (GenBank accession number KR_610991); Vietnam (GenBank accession number KJ_960179); Korea (GenBank accession number KJ_662670).

Madhugiria et al. showed that the ≈310 nts of the 5′ terminal genome region of *Alphacoronavirus* form four secondary structures: SL1, SL2, SL4, and SL5 [4]. Accordingly, the sequence representing the 300 nts of the 5′ terminal region obtained for PEDV-positive samples (TW strain) were analyzed and compared with that of the PEDV prototype CV777. To understand whether the sequences for the 5′ terminal genome region of other PEDV strains are also altered, the reference strains were also selected from GenBank based on the viruses that have been circulating in different countries in recent years (2012–2015) and compared. As shown in Figure 1, in comparison with CV777, a U-insertion at nt 48; an A deletion at nt 73, which was located immediately downstream of the TRS core sequence (CUAAAC); and a 4-nt deletion (UUCC) from nt 93 to 96 were identified. In addition, the bases at positions 8, 104, 122, and 292 were also altered to a G, C, A, and U, respectively. Of the PEDV reference strains selected from other countries, the 5′-terminal sequence for the TW strain shared 100% nucleotide identity with that of the USA, Germany, and Korea reference strains. The change of the C and A at positions 104 and 122, respectively, also altered the amino acid encoded by the uORF (Figure 1B,C). Using the Mfold algorithm [36], four SLs were identified in the 5′ terminal genome region of PEDV and the results were in agreement with those found by Madhugiria et al. in *Alphacoronaviruses* [4]. In comparison with CV777, the change at nt 8 for the TW strain slightly altered the structure of SL1 (Figure 1C), and the U-insertion at nt 48 slightly altered the loop but not the stem structure of SL2. The free energy (∆G) of SL1 and SL2 was found to increase from −11.1 and −1.8 kcal/mol for the CV777 strain to −12.5 and −3.9 kcal/mol for the TW strain, respectively. The 4-nt deletion plus 2 alterations in SL4 also slightly affected the overall structure of SL4, and a decrease in ∆G from −27.4 for CV777 strain to −24.3 kcal/mol was calculated for TW strain. Although the U-alteration at nt 292 did not impair the structure of SL5, ∆G decreased from −53.2 to −51.6 kcal/mol. The overall ∆G of the four structures for CV777 and Taiwan strain was found to be −97.3 and −96.2 kcal/mol, respectively.

### 3.2. Comparison of 3′ UTR Sequence and Structure between CV777 and TW Strains

**Figure 2:**
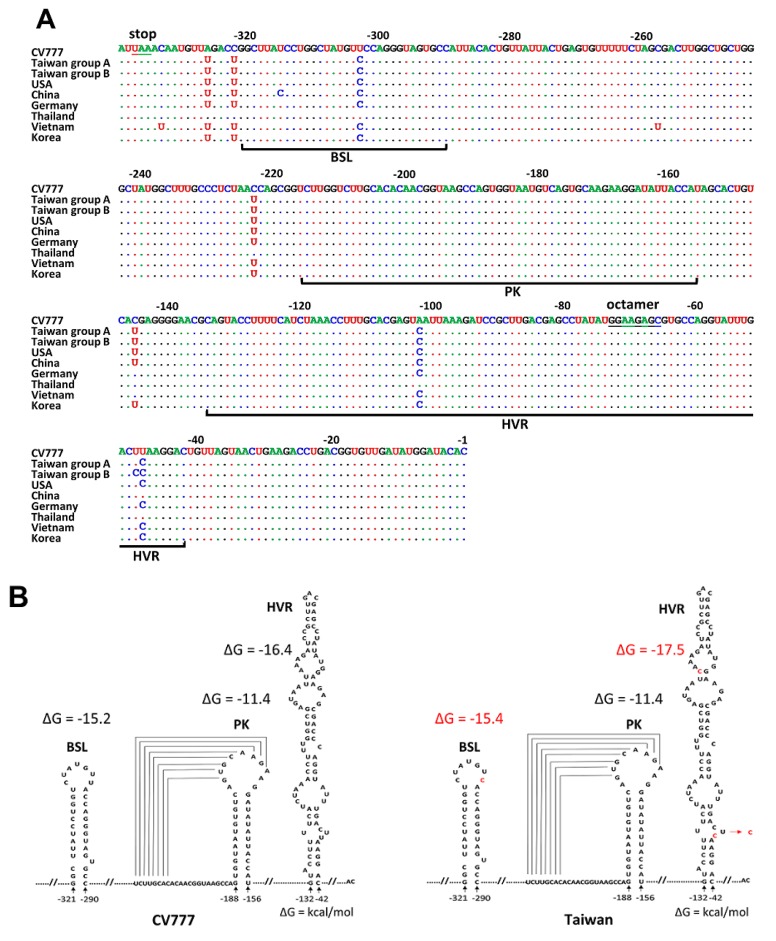
Sequence and structures of the PEDV 3′ UTR. (**A**) Nucleotide sequence alignment of the 3′ UTR between CV777, TW, and reference strains. The UAA stop codon for the N ORF and a highly conserved octarmeric element, GGAAGAGC, are underlined. The position numbers, counting from the poly(A) tail, refer to the composite sequence. Nucleotide identity between sequences is indicated with a dot; a difference is indicated with a new letter code. (**B**) Structure prediction of the 3′ UTR of CV777 and TW. Please refer to Figure 1 for the GenBank accession numbers of the reference strains. BSL, bulged stem-loop; PK, hair-pin pseudoknot; HVR, hypervariable region.

Using a sequencing analysis of 3′ UTR, either U or C at nt −49 was found from PEDV-positive samples (TW strain). The PEDV TW strain was therefore divided into two groups: Taiwan group A (with U at nt −49) and Taiwan group B (with C at nt −49) (Figure 2A). Regarding 3′ UTR, 7- and 8-nt differences between CV777 and TW groups A and B, respectively, were identified (Figure 2A). TW strain group A shared 100% nt identity with the USA and Korea strains. The C at nt −49 in the TW group B is unique because it was not identified in any sequenced PEDV genome, as based on data from GenBank; thus, it can be used as a genetic marker to differentiate the TW strain from other PEDV strains. Using the Mfold algorithm [36], the BSL, PK, and HVR structures were predicted (Figure 2B). Of the nts that are different from those of CV777, one nt is located in the BSL structure, and three nts (TW strain group A) and four nts (TW strain group B) are located in the HVR structure. However, these changes do not alter the overall structure of BSL and HVR; the ∆G for both structures was found to be slightly increased in comparison with that for CV777.

### 3.3. Identification of a Novel Subgenomic mRNA Species Derived from the 3′ UTR

**Figure 3:**
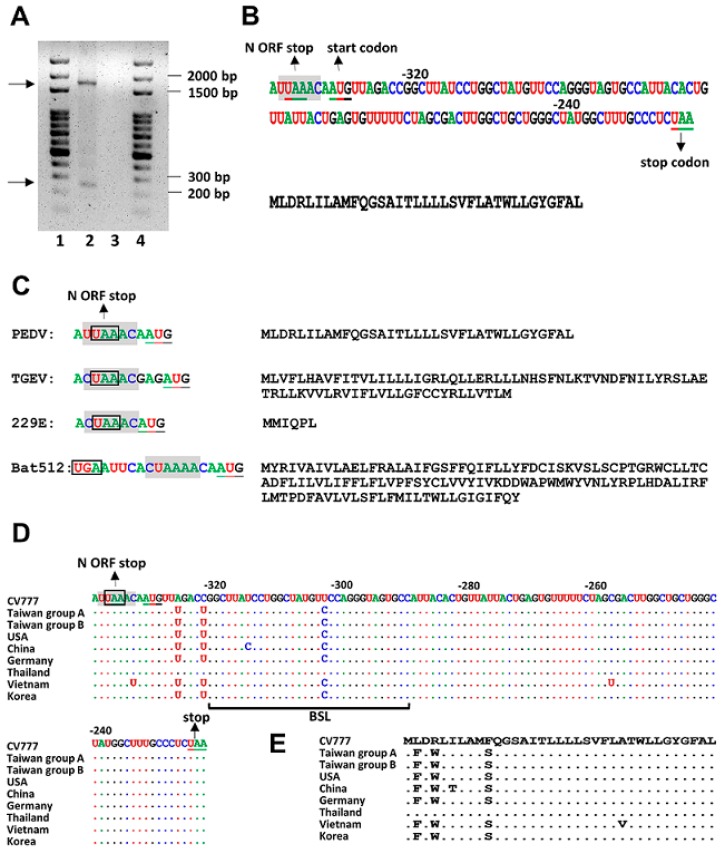
Identification of a novel subgenomic mRNA (sgmRNA) derived from the 3′ UTR of PEDV. (**A**) Identification of a sgmRNA derived from 3′ UTR. RT-PCR products of ≈1700 bp and ≈250 bp were observed from PEDV-infected Vero cells (lane 2), but not from mock-infected Vero cells (lane 3). Lanes 1 and 4, ds DNA size markers. (**B**) Upper panel: Nucleotide sequence of the newly identified sgmRNA ORF. The stop codon for the N ORF is boxed. CS-B is shaded in gray. The potential start codon and stop codon for the ORF are underlined. Lower panel: The deduced amino acid sequence of the ORF shown in upper panel. bp, base pair. (**C**) Left panel: Illustration of the relative position of the stop codon (for the N ORF), core sequence (for initiating the corresponding sgmRNA) and start codon of the ORF for PEDV, transmissible gastroenteritis coronavirus (TGEV), coronavirus 229E, and bat coronavirus 512. The stop codon for the N ORF is boxed. CS-B is shaded in gray. The start codon for the ORF is underlined. Right panel: Deduced amino acid sequence of the ORF for the aforementioned *Alphacoronaviruses*. (**D**) Alignment of the nucleotide sequence of the ORF between CV777, TW, and reference strains. CS-B is shaded in gray. The stop codon for the N ORF is boxed. The start and stop codons for the novel ORF is underlined. (**E**) Alignment of the deduced amino acid sequence of the novel ORF between CV777, TW, and reference strains. TGEV (GenBank accession number DQ811788.1); coronavirus 229E (GenBank accession number JX503061.1); Scotophilus bat coronavirus 512 (GenBank accession number NC_009657.1).

While analyzing the 3′ UTR sequences of TW strain, with the primers annealing the 5′ leader sequence and 3′ UTR, the reverse transcription polymerase chain reaction (RT-PCR) products with a length of ≈1700 base pairs (bp) and ≈250 bp were observed (Figure 3A, lane 2). After sequencing analysis, the ≈1700 bp RT-PCR product was identified to be N sgmRNA and the ≈250 bp RT-PCR product, which consisted of a leader sequence and a part of the sequence from 3′ UTR, was a previously unidentified sgmRNA species. The sgmRNA was derived from the CS-like sequence UUAAAC, which encompassed the stop codon of the N gene (Figure 3B, upper panel). The potential start codon AUG of the sgmRNA, which was located one nt downstream of the CS-like sequence, was also identified (Figure 3B, upper panel), and the ORF was predicted to encode a protein of 35 amino acids (Figure 3B, lower panel). In TGEV, sgmRNA 7 was also derived from a CS at a genome position similar to that in PEDV (Figure 3C), and the encoded protein of 78 amino acids has been demonstrated to be involved in the virulence [37]. Such a CS sequence at a position similar to that PEDV was also found in coronavirus 229E and Scotophilus bat coronavirus 512 (Figure 3C), though the sgmRNAs derived from both putative CSs have not been experimentally identified. In comparison with CV777, three nts and three amino acids were altered within the ORF (Figure 3D,E, respectively). The region downstream of the gene encoding the N protein shows variations among *Alphacoronaviruses*, which may explain why the conserved counterpart of the *Betacoronavirus* BSL structure [9,10] could not be identified as a common structure in *Alphacoronaviruses* [4]. For PEDV, the BSL structure was identified using the Mfold algorithm [36], as shown in Figure 2B, yet it was located within the putative ORF, as described above (Figure 3D). The function of the protein encoded by the ORF of the sgmRNA and the importance of the predicted BSL structure to PEDV replication remain to be elucidated.

### 3.4. Effects of the *Cis*-Acting Element Variations between CV777 and TW Strains on Gene Expression

**Figure 4:**
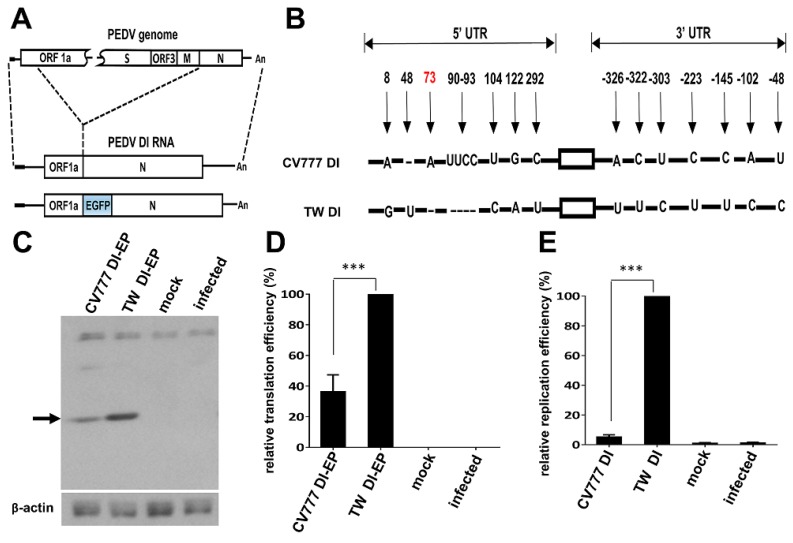
Effects of variations in the 5′ and 3′ UTRs between CV777 and TW strains on translation and replication. (**A**) Diagram showing the genome structure of the PEDV TW strain, PEDV defective interfering (DI) RNA, and PEDV DI RNA with the *EGFP* gene. (**B**) Illustration of sequence variations in both the 5′ and 3′ UTRs between the PEDV Taiwan strain DI RNA (TW DI) and CV777 strain DI RNA (CV777 DI). (**C**) Detection of translation from TW DI-EP and CV777 DI-EP in Vero cells using western blotting with an antibody against EGFP. (**D**) Relative translation efficiency between TW DI-EP and CV777 DI-EP, as based on the results shown in (C). (**E**) Relative replication efficiency between TW DI and CV777 DI, as quantitated by RT-qPCR. The values in (D) and (E) represent the means ± standard deviation (SD) of three individual experiments. *** *p* < 0.001.

Coronavirus DI RNA has been intensively employed for gene expression analyses [38,39,40,41,42]. To determine whether the observed variations in the 5′ and 3′ UTRs between TW and CV777 strains alter the efficiency of gene expression, a PEDV DI RNA for the TW strain was constructed; it was designated TW DI (Figure 4A). Because the CV777 strain was not available due to regulations in Taiwan, a CV777 DI RNA (designated CV777 DI, Figure 4B) was constructed via mutagenesis, which had TW DI as a backbone but with the CV777 5′ and 3′ UTRs. For translation analysis, both DI RNAs were engineered to contain a reporter *EGFP* gene (Figure 4A) designated CV777 DI-EP and TW DI-EP. Vero cells were transfected with 3 µg of in vitro-transcribed RNA, and proteins in cell lysates were collected at 16 h posttransfection. The amount of synthesized EGFP was normalized to the levels of internal controls including β-actin and DI RNA. Note that both the DI RNAs cannot replicate because of the lack of helper virus in transfected cells and the stability of the two DI RNAs was similar at the time of RNA collection (data not shown). As shown in Figure 4C,D, the translation efficiency of TW DI-EP was ≈2.5-fold better than that of CV777 DI-EP, suggesting that the sequence alterations in the 5′ and 3′ UTRs of TW DI-EP increased the translation efficiency in comparison with that of strain CV777. Note that, because uORF has been suggested to have an influence on translation, in addition to alterations in sequence and structure at the 5′ and 3′ UTRs, the amino acid changes for uORF (Figure 1) may be a factor leading to the observed difference in translation efficiency.

Regarding the effect on replication, the replication efficiency for CV777 DI was extremely low when compared with that for TW DI (≈20-fold difference) (Figure 4E) after 48 h of VP1. Sequencing analysis revealed that the leader sequence in CV777 DI was, in part, replaced by that of the helper virus TW strain, as a G at nt 8 and a U at nt 48 were identified. Except for the alterations in the leader sequence, no other changes in CV777 DI were found. It has been demonstrated that leader-switching occurred with a high frequency in the TRS region between DI RNA and the helper virus [43,44], and this may explain the replacement of the nts that occurred in the CV777 DI RNA leader sequence. Because it has been suggested that DI RNA is able to compete and recombine with the helper virus, such a nature may complicate the interpretation of the DI replication assay [45]. In the current study, it was found that the leader sequence of CV777 DI RNA was replaced by that of the helper virus, and consequently the recombination event may affect the quantitative results of the DI RNA replication assay. Whether the replication efficiency between wild-type CV777 and TW strain with full-length genome still shows a dramatic difference remains to be determined. Accordingly, at this point, we can only conclude that the variations in 5′ and 3′ UTRs between CV777 and TW may influence replication efficiency using the DI RNA system. Further study using PEDV reverse genetic system is required to determine to what extent the variations in 5′ and 3′ UTRs between the two strains affect replication efficiency.

### 3.5. Analyses of the TRS for CV777 and TW Strain

**Figure 5:**
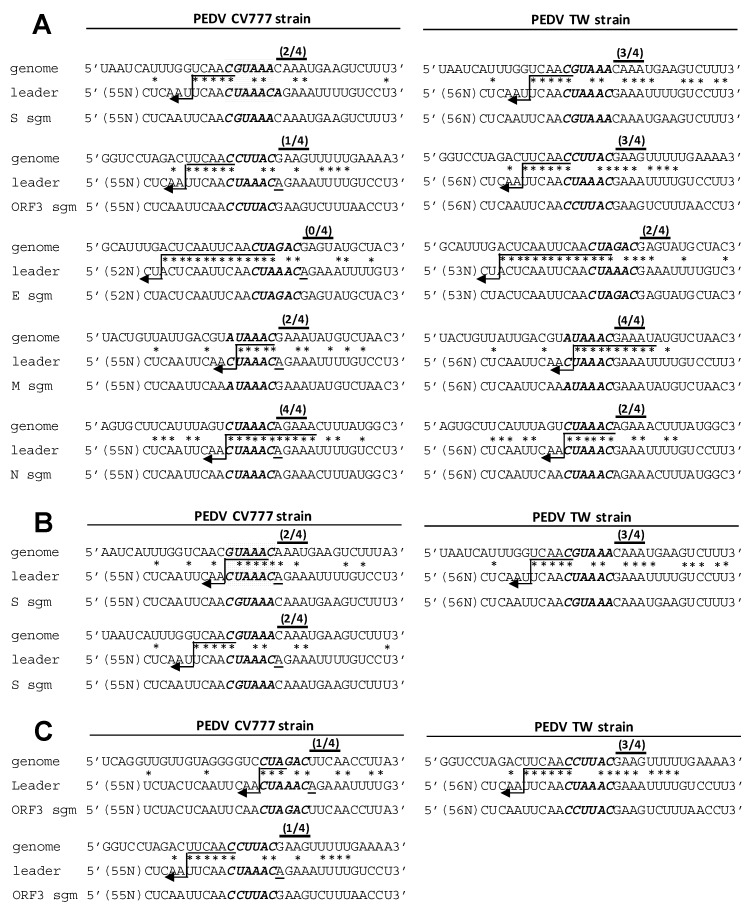
Alignment of identified fusion sites in the genome with the 5′ end of the CV777 strain (left panel) or TW strain (right panel). CS-B and CS-L are identified by shading. The identity of clustered base similarities within the 14-nt region (including the 4-nt 5′-TRS, 6-nt CS-B, and 4-nt 3′-TRS) immediately surrounding the CS-B is also shown. Nucleotide identity between sequences is indicated using an asterisk. The number of nt identity among the 4-nt 3′-TRS is indicated above the 3′-TRS. Postulated polymerase strand-switching during minus-strand synthesis is indicated by an arrow. Because the CS-B for the *S* and *ORF3* genes for CV777 are unknown, the currently assumed CS-B for the two genes is shown in the left panel of (**A**). The previously (upper left panel) and currently (lower left panel) assumed CS-B for the *S* gene and *ORF3* genes for CV777 is also used for the alignment, as shown in the left panel of (**B**) and (**C**), respectively.

TRSs are *cis*-acting elements required for coronavirus sgmRNA synthesis that are located at the 3′ end of the leader sequence (TRS-L) and preceded by each gene (TRS-B). The TRS contains a CS flanked by variable sequences at its 5′ (5′ TRS) and 3′ (3′ TRS) ends of the CS. The CS of TRS-L (CS-L) in PEDV CV777 is CUAAAC. The CS of TRS-B (CS-B) for CV777 strain *E*, *M*, and *N* genes has been experimentally determined to be CUAGAC, AUAAAC, and CUAAAC, respectively [46] (Figure 5A, left panel for *E*, *M*, and *N* genes). Although the CS-B for the *S* and *ORF3* genes for CV777 strain was previously assumed to be GUAAAC and CUAGAC, respectively [47] (Figure 5B,C, respectively, upper left panel), this has yet to be experimentally determined. In comparison with CV777, the CS-L of the TW strain was not altered, i.e., it was CUAAAC (Figure 1A). To determine the CS-B for TW, sgmRNA was amplified using RT-PCR followed by sequencing. The sequencing results were analyzed to identify the fusion sites in the genome with the 5′ end of the virus, by which the CS-B for the *S*, *ORF3*, *E*, *M*, and *N* genes was determined (Figure 5A, right panel). The results suggested that the CS-B for *E*, *M*, and *N* was the same for PEDV TW and CV777; however, the CS-B for *S* and *ORF3* for TW was different from the previously assumed CS-Bs for CV777. Instead of the previously assumed GUAAAC for CV777 (Figure 5B, upper left panel), the CS-B for the TW *S* gene was CGUAAA (Figure 5B, upper right panel), which was located one nt upstream of previously assumed GUAAAC. Based on the study by Sola et al. that found that TRS is defined as consisting of the central CS and the four nts immediately 5′ (5′ TRS) and 3′ (3′ TRS) of the CS, the overall identity of the *S* TRS-B with TRS-L for CV777 was slightly lower than that for TW (8 for CV777 and 10 for TW; Figure 5B, upper panel). We speculate the reason why GUAAAC was previously assumed to be the CS-B for the CV777 *S* gene may be attributed to its higher sequence identity with CS-L in comparison with CGUAAA for TW (5 for CV777 and 3 for TW; Figure 5B, upper panel). However, the higher identity between the 5′ TRS-B and 5′ TRS-L (1 for CV777 and 4 for TW) and between the 3′ TRS-B and 3′ TRS-L (2 for CV777 and 3 for TW) may be a more decisive factor for the outcome based on CGUAAA being selected for TW. With regard to *ORF3*, CS-B CCUUAC for TW (Figure 5C, upper right panel), which was located 11 nts downstream of the previously assumed CUAGAC for CV777 (Figure 5C, upper left panel), was identified. The same reason as described above for the *S* gene CS-B may be involved, whereby the previously assumed CUAGAC of *ORF3* for CV777 showed higher identity with the CS-L in comparison with that for TW while lower identity between the 5′ TRS-B and 5′ TRS-L (0 for CV777 and 4 for TW) and between the 3′ TRS-B and 3′ TRS-L (1 for CV777 and 3 for TW) is observed. Based on the argument above, it is currently assumed that the CS of TRS-B (CS-B) for CV777 strain *S* and *ORF3* genes is CGUAAA (Figure 5A, left panel and Figure 5B, lower left panel) and CCUUAC (Figure 5A, left panel and Figure 5C, lower left panel), respectively; however, the exact CS-B of *S* and *ORF3* genes for CV777 remains to be experimentally determined with RNA collected from CV777-infected cells.

### 3.6. Evaluation of the Effect of Variations in TRS between CV777 and TW Strains on sgmRNA Synthesis

According to the current study shown in Figure 1 and previous reports [3,18,19], in comparison with CV777, an A deletion at nt 73 immediately downstream of CS-L was frequently identified for PEDV, including the TW strain. It has been proposed that such a deletion immediately downstream of CS-L may have influence on PEDV sgmRNA synthesis [3,18,19], though further examination to support this hypothesis has been not performed. As the TRS is defined to consist of the central CS and the four nts immediately flanking the 5′ (5′ TRS) and 3′ (3′ TRS) CS [17] and based on the alignment shown in Figure 5A, the one-nt deletion immediately downstream of the CS-L in the TW strain (Figure 5A, right panel) results in increased identity for the 3′ TRS between TRS-B and TRS-L for *S*, *ORF3*, *E*, and *M*, but not *N* genes, in comparison with CV777 (Figure 5A, left panel). This was also true for comparison of the previously (Figure 4B,C, upper left panel) and currently (Figure 4B,C, lower left panel) assumed CS-B of *S* and *ORF3* gene for CV777. Because the 3′ TRS has a more decisive influence on template-switching during sgmRNA synthesis [16,17], the increased identity of the 3′ TRS in these genes may enhance sgmRNA synthesis. Furthermore, it has been suggested that if the identity of the 5′ TRS and 3′ TRS of CS-B with those of CS-L reaches a plateau in *G* value, template-switching is able to occur for sgmRNA synthesis, even though CS-B exhibits low identity with CS-L [17]. Thus, in comparison with the TW strain, the decreased identity in the CV777 3′ TRS may have an influence on the synthesis of *S*, *ORF3*, and *M* sgmRNAs because their CS-Bs also have low identity with CS-L. Accordingly, in comparison with CV777, the increased sequence identity of the 3′ TRS between TRS-L and TRS-B of *S*, *ORF3*, *E*, and *M* (but not *N*) genes in TW may alter the relative efficiency of sgmRNA synthesis.

**Figure 6:**
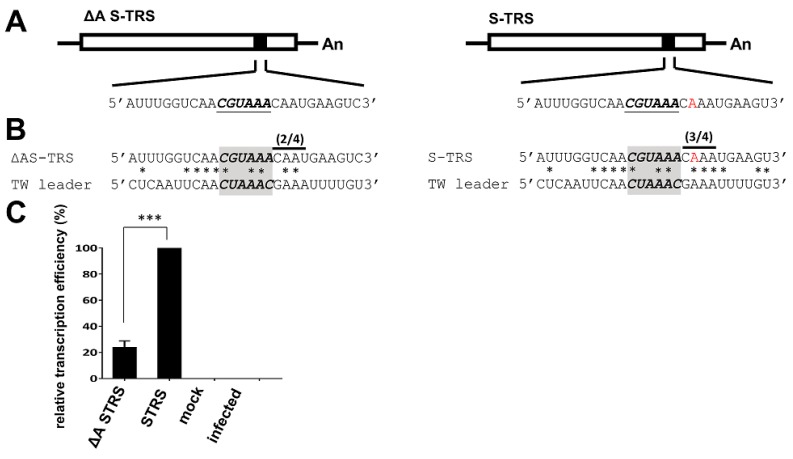
Effect of sequence identity between TRS-L and TRS-B on the efficiency of sgmRNA synthesis. (**A**) Genome structure of the TW DI RNA with an insertion of the TRS for *S* gene. In constructing ΔA S-TRS, an A residue in the 3′ TRS was deleted (left panel); however, in constructing S-TRS, no deletion at the same position was created (right panel). (**B**) Based on sequence alignment, deletion of the A residue from the 3′ TRS led to 2-nt identity of the 3′ TRS between TRS-L and TRS-B (left panel). In contrast, without the A residue deletion, three nts of the 3′ TRS are identical between TRS-L and TRS-B in constructing S-TRS (right panel). (**C**) Relative efficiency of sgmRNA synthesis between ΔA S-TRS and S-TRS, as quantitated by RT-qPCR. The values in C represent the means ± SD of three individual experiments. *** *p* < 0.001.

To determine whether the altered sequence identity between the leader TRS and body TRS (Figure 5) affected sgmRNA synthesis within the same context of the genome, the TRS-B for the *S* gene was inserted into TW DI (designated S-TRS; Figure 6A, right panel). In addition, ΔA S-TRS (Figure 6A, left panel) was also constructed, in which the 3′ TRS of the TRS-B for the *S* gene was mutated to decrease its sequence identity with the 3′ TRS of the TRS-L (Figure 6B). As shown in Figure 6C, the efficiency of subgenomic DI RNA synthesized from ΔA S-TRS was decreased in comparison with that from S-TRS. The results suggest that the increase in sequence identity between the leader TRS and body TRS leads to enhanced subgenomic mRNA synthesis, which is in agreement with our hypothesis. Indeed, these results were not unexpected based on previously published studies regarding the correlation of TRS sequence identity with sgmRNA synthesis [16,17,48,49,50]. However, it is the first study on the content of a PEDV genome demonstrating that the increased sequence identity between the leader TRS and body TRS enhances sgmRNA synthesis. The results also support the previously proposed hypothesis [3,18,19] that the increased 3′ TRS sequence identity between TRS-L and TRS-B in the TW strain caused by a deletion immediately downstream of CS-L has influence on PEDV sgmRNA synthesis. In conclusion, the results support our hypothesis that increased 3′ TRS sequence identity between TRS-L and TRS-B in the TW strain caused by a deletion immediately downstream of CS-L has an influence on PEDV sgmRNA synthesis.

Overall, we have determined that the *cis*-acting elements were altered in the PEDV TW strain and the reference strains (except the Thailand strain) in comparison with those of the PEDV prototype strain CV777. Functional analyses suggest that such variations in *cis*-acting elements for the TW strain compared with the CV777 strain have an influence on the efficiency of gene expression. Therefore, it is speculated that variations in *cis*-acting elements may also be one of the factors potentially contributing to the pathogenesis. Although the *cis*-acting elements of the Thailand reference strain are the same as those of the CV777 strain, we still cannot exclude the importance of the *cis*-acting elements because it is not clear whether the Thailand reference strain is virulent or not based on the insufficient information from GenBank. Accordingly, we also cannot rule out other factors that also play roles in the PED outbreak. Further study is required to determine to what extent the variations in *cis*-acting elements affect the virulence and pathogenesis using a reverse genetic system.

## 4. Conclusions

In this study, we found variations in *cis*-acting elements in the 5′ and 3′ UTRs between the PEDV TW and prototype CV777 strains. We also determined the previously unidentified *cis*-acting element TRS and found that (i) a nucleotide deletion in the TW strain, in comparison with CV777 strain, immediately downstream of the leader core sequence alters the identity between the leader TRS and the body TRS, and (ii) the altered sequence identity has influence on coronavirus subgenomic mRNA synthesis. Functional analyses revealed that such variations in the 5′ and 3′ UTRs of the TW strain also alter the efficiency of gene expression in comparison with that of the CV777 strain. Because the analyzed *cis*-acting elements for the TW strain are the same as those of the reference strains for USA and Korea, our results may extend our understanding of PEDV evolution in terms of *cis*-acting elements and the recent outbreaks of PED worldwide. Further study using reverse genetic system is required to determine whether the altered gene expression is associated with the increased severity of the recent PED outbreaks. In addition, a putative ORF within the 3′ UTR and a BSL structure within the putative ORF were also found. The biological significance of the ORF and BSL structure remains to be experimentally demonstrated.

## Supplementary Materials

The following are available online at http://www.mdpi.com/2073-4425/9/12/591/s1, Table S1: Oligonucleotides used for this study.
