# Supplementary material for: Gene Variations in Cis-Acting Elements between the Taiwan and Prototype Strains of Porcine Epidemic Diarrhea Virus Alter Viral Gene Expression
Source: Genes (Basel). 2018 Nov 29;9(12):591. doi: 10.3390/genes9120591 (PMC6316102; doi:10.3390/genes9120591)
Supplement: Supplementary file 1 [file genes-09-00591-s001.pdf]

Table S1. Oligonucleotides used for this study

Oligonucleotide	Sequence
PEDV 49T deletion (-)[a]	5' TAgCTCTTTTTCTAgACCTTgTCTACTCAATTCAACTAA 3'
73A addition (-)	5' gTCTACTCAATTCAACTAAACAgAAATTTTgTCCTTCCggC 3'
PEDV 90-93 TTCC addition (-)	5' AATTTTgTCCTTCCTTCCggCCgCATgTCCATgCTgCTgg 3'
C104T Mutation (-)	5' gTCCTTCCggCCgCATgTTCATgCTgCTggAAgCTgACgT 3'
A122G Mutation (-)	5' ATgCTgCTggAAgCTggCgTggAATTTCATTAggTTTgCT 3'
T292C Mutation (-)	5' TgAAACCAgTAACTgCCggCTATggCTAgCAACCATgTTA 3'
3' UTR-C48T-T49C (-)	5' gCgTgCCAggTATTTgACTTAAggACTgTTAgTAACTg 3'
3' UTR-C99T (-)	5' CCTTTgCACgAgTAATTAAAgATCCgCTTgACgAgCC 3'
3' UTR-T145C (-)	5' ggATATTACCATAgCACTgTCACgAggggAACgCAgTACC 3'
3' UTR-T222C (-)	5' gCTATggCTTTgCCCTCTAACCAgCggTCTTggTCTTgC 3'
3' UTR-C303T (-)	5' gCTTATCCTggCTATgTTCCAgggTAgTgCCATTACACTg 3'
3' UTR-T322C-T326A (-)	5' ggTTgCTAAATAAACAATgTTAgACCggCTTATCCTggCTA 3'
PEDV C57T (-)	5' CTAgACCTTgTCTACTCAATTCAACTAAAC 3'
PEDV C862T (-)	5' ggTTgCTAAATAAACAATgTTAgACCggCTTATCCT 3'
PEDV G921A (-)	5' ACACTgTTATTACTgAgTgTTTTTCTAgCgAC 3'
PEDV T1019C (-)	5' gTggTAATgTCAgTgCAAgAAggATATTACC 3'
PEDV A1086G (-)	5' CATCTAAACCTTTgCACgAgTAATTAAAgATCC 3'
PEDV C1118T, C1128T (-)	CTTgACgAgCCTATATggAAgAgCgTgCCAggTATTTgAC 3'
PEDV 5'UTR-EGFP (-)	5′ CCAAgCTTAgTgCgTATgTggACACTATggTgAgCAAgggCgAggAgCTgTTC 3′
EGFP (+)	5′ CTTgTACAgCTCgTCCATgCCgAg 3′
PEDV 5'GD (-)	5′ CAAATATgCCTTggCggATgCCTCCAgCAgCATgAAgAggCC 3′
mut PE-sis2 (-)	5′ gATTggTCAACgTAAACAATgAAgTCTTTCCAAATgTTgAgCTTC 3′
T7-PEDV 5'UTR(-)	5′ TgTAATACgACTCACTATAgggACTTAAAAAgATTTTCTATCTACgg 3′
PEDV 25 (-)	5' ACTTAAAAAgATTTTCTATCTACgg 3'
PEDV 476 (-)	5' gTCATggTggTggTCggCAC 3'
PEDV SIS (-)	5' TggTCAACgTAAACAAATgAAgTCTTTCCAAATgTTgAgCTTC 3'
PEDV 26541 (-)	5′ gAgCAAATTCgCTggCgCATgCgCCgTggTg 3′
PEDV 27488 (-)	5' gAgCTTCTTgTTTCACAggTgg 3'
PEDV 450 (+)	5' TgTCAgCgAgATCgAgggAC 3'
PEDV ORF3 (+)	5' TTgCgCCTCAAAgAAgACgC 3'
PEDV S 20874 (+)	5' gATACCATgAACgCCACTAgCAg 3'
PEDV M 25831 (+)	5' CTTgACACCATACAAgAACgC 3'
PEDV E 25674 (+)	5' gTCAATAACAgTACTggggAggg 3'
PEDV N 26538 (+)	5' CCAgTACCAATTTgCTggTCC 3'
PEDV 26696 (+)	5′ TTCAgTCTTTgCgCCTTCTTTAgCAACCCAgAAAAC 3′
PEDV 3'utr (+)	5' gTgTgcAAgACCAAgACCgCTg 3'
PEDV 65A(+)	5' T (65)gTgTATCCATATCAACACCg 3'
TGEV R (-)	5' TgCCAAggATggTgCCATggATCggAATgAgCA 3'
TGEV(+)	5' gATCCATggCACCATCCTTggCAACCCAgA 3'

a..The positive and negative symbols in the oligonucleotide names indicate the polarities of the nucleic acids to which the oligonucleotides anneal. Oligonucleotides named with a negative symbol used for mutagenesis in the text have a sequence complementary to a positive-sense oligonucleotide of the same name.
